# Particulate Air Pollution, Progression, and Survival after Myocardial Infarction

**DOI:** 10.1289/ehp.9201

**Published:** 2007-02-20

**Authors:** Antonella Zanobetti, Joel Schwartz

**Affiliations:** Department of Environmental Health, Harvard School of Public Health, Boston, Massachusetts, USA

**Keywords:** air pollution, epidemiology, heart diseases, myocardial infarction, survival

## Abstract

**Objective:**

Several studies have examined the effect of particulate pollution (PM) on survival in general populations, but less is known about susceptible groups. Moreover, previous cohort studies have been cross-sectional and subject to confounding by uncontrolled differences between cities.

**Design:**

We investigated whether PM was associated with progression of disease or reduced survival in a study of 196,000 persons from 21 U.S. cities discharged alive following an acute myocardial infarction (MI), using within-city between-year exposure to PM. We constructed city-specific cohorts of survivors of acute MI using Medicare data between 1985 and 1999, and defined three outcomes on follow-up: death, subsequent MI, and a first admission for congestive heart failure (CHF). Yearly averages of PM_10_ (particulate matter with aerodynamic diameter < 10 μm) were merged to the individual annual follow-up in each city. We applied Cox’s proportional hazard regression model in each city, with adjustment for individual risk factors. In the second stage of the analysis, the city-specific results were combined using a meta-regression.

**Results:**

We found significant associations with a hazard ratio for the sum of the distributed lags of 1.3 [95% confidence interval (CI), 1.2–1.5] for mortality, a hazard ratio of 1.4 (95% CI, 1.2–1.7) for a hospitalization for CHF, and a hazard ratio of 1.4 (95% CI, 1.1–1.8) for a new hospitalization for MI per 10 μg/m^3^ PM_10_.

**Conclusions:**

This is the first long-term study showing a significant association between particle exposure and adverse post-MI outcomes in persons who survived an MI.

Studies have shown short-term effects of particulate pollution (PM) on hospital admissions and deaths from cardiovascular causes (Anderson et al. 2003; Braga et al. 2001; Dockery 2001; Katsouyanni et al. 1996; Pope et al. 2004a; Samet et al. 2000; Schwartz 1999; Zanobetti et al. 2000). Myocardial infarctions (MIs) have been shown to be susceptible to being triggered by PM (Braga et al. 2001; D’Ippoliti et al. 2003; Lanki et al. 2006; Peters et al. 2001a; von Klot et al. 2005; Zanobetti and Schwartz 2005). These studies have not addressed whether persons who survive an MI are at risk of death in response to subsequent particle exposure.

A few studies have addressed this question with respect to acute exposure. For example, Bateson and Schwartz (2004) reported subjects in Chicago who were discharged alive for an MI had twice the risk of death due to acute air pollution exposure as other subjects.

In a recent study of five European cities, von Klot et al. (2005) found that ambient air pollution was associated with increased risk of hospital cardiac readmissions of MI survivors. Peters et al. (2006) examined the probability of recurrent hospitalization in a cohort of MI survivors; compared the use of time-series, case–crossover, and survival analysis for analyzing short-term health effects; and found that the three methods gave similar results. None of these studies examined the effect of longer-term exposure on survival.

Several studies have examined the effect of longer-term PM exposure on survival in general cohorts. The Harvard Six Cities Study (Dockery et al. 1993) demonstrated an association between mortality and chronic exposure to PM. A recent reanalysis of the Harvard Six Cities Study, which extended the mortality follow-up period, used a similar approach to our study and included PM_2.5_ (PM with aero-dynamic diameter < 2.5 μm) as a time-varying exposure; Laden et al. (2006) found significant association of PM_2.5_ with mortality.

Two articles (Pope et al. 2002, 2004a) have shown an association between PM and mortality in the American Cancer Society (ACS) Cancer Prevention Study population, including an association between PM and deaths from all cardiovascular disease (Pope et al. 2004a).

These studies have treated air pollution as a city-level variable, whereas two European cohort studies have assessed exposure at an individual level. Hoek et al. (2002) found an association between estimated long-term exposure to traffic-related particles at each participant’s home and cause-specific mortality in the Netherlands Cohort Study on Diet and Cancer. Nafstad et al. (2004) found an association between estimated nitrogen dioxide concentrations at the subject’s home and the risk of dying for total, respiratory, lung cancer, and ischemic heart disease mortality in a cohort of men in Oslo, Norway. Particle data was not available in that study, and NO_2_ was used as a marker of traffic pollution.

Although these studies have reported an association of PM with survival, they have not evaluated the role of preexisting cardiac disease or whether risk changed with annual changes in exposure, nor have they treated pollution as a time-varying covariate.

Reduced heart rate variability has been associated with decreased survival of MI patients (Ewing 1991; Malik et al. 1989), and PM has been associated with reduced heart rate variability (Creason et al. 2001; Gold et al. 2000; Liao et al. 1999; Park et al. 2005; Pope et al. 1999), which in turn is associated with decreased post-MI survival (Stein et al. 2005). Other studies have suggested that PM may be associated with increased C-reactive protein (Brook et al. 2003; Peters et al. 2001b), which is associated with mortality risk following an MI (Kinjo et al. 2005). PM and its components have also been shown to increase oxidative stress in the heart (Bouthillier et al. 1998; Brook et al. 2003; Dhalla et al. 2000; Sorensen et al. 2003), to decrease plaque stability (Suwa et al. 2002), and to increase atherosclerosis (Künzli et al. 2005). Based on this, we investigated whether annual PM exposure was associated with progression of disease or reduced survival in a study of 196,000 persons discharged alive following an acute MI.

## Materials and Methods

### Study population

Using Medicare data for persons ≥ 65 years of age, we constructed a cohort of survivors of acute MI, defining cases as emergency admissions for a primary discharge diagnosis of MI [*International Classification of Diseases, Ninth Revision* (ICD-9; World Health Organization 1975) code 410] discharged alive between 1985 and 1999 in any of 21 cities chosen to represent a broad range of the country. We obtained from Medicare the date of death for each subject, or whether they were still alive as of the end of 1999. We also retrieved information on age, sex, race, and the number of coronary intensive care days and Medical intensive care days.

To study progression of disease, we traced each subject through subsequent Medicare records and identified admissions for a subsequent MI or for congestive heart failure (CHF; ICD-9 code 428).

Subjects alive the first of January of the year following the admission were entered into the cohort, and follow-up periods were calendar years. We excluded subjects whose death or subsequent admission occurred within the first 3 months of their index admission.

We also used a unique identifier for each subject to assess medical factors that might modify the risk of survival or progression, such as whether they had any primary or secondary diagnosis of chronic obstructive pulmonary disease (COPD; ICD-9 codes 490–496, except 493), diabetes (ICD-9 code 250), or essential hypertension (ICD-9 code 401); or if they had previous admissions for atrial fibrillation (ICD-9 code 427.3).

During 1985–1999 changes in treatment occurred, such as introduction of thrombolytics and increased angioplasty. To control for changes in postdischarge survival, we used strata to allow a different underling hazard for each 5-year interval in the study.

We defined a categorical variable for type of MI as given by the fourth digit of the ICD-9 code. There are 10 different types of MI: ICD-9 code 410.0, MI of anterolateral wall; 410.1, other anterior wall; 410.2, inferolateral wall; 410.3, inferoposterior wall; 410.4, other inferior wall; 410.5, other lateral wall; 410.6, true posterior wall infarction; 410.7, subendocardial infarction; 410.8, other specified sites; and 410.9, unspecified sites.

City characteristics, such as population density and percentage of population ≥ 65 years of age in poverty status, were obtained from the 1990 U.S. census (U.S. Census Bureau 2000). The average annual mortality rate for emphysema among people ≥ 65 years of age during 1989–2000 were obtained from the National Center for Health Statistics (Hyattsville, MD) and used as an indirect measure of smoking history in each city.

The accuracy of the Medicare claims-based diagnosis of MI has been recently validated (Kiyota et al. 2004).

### Environmental data

We obtained data for PM_10_ (particulate air matter with aero-dynamic diameter < 10 μm) for 1985–1999 from the U.S. Environmental Protection Agency’s Aerometric Information Retrieval System (Nehls and Akland 1973).

We selected the following cities with daily PM_10_ monitoring that represent a geographic distribution across the country: Birmingham, Alabama; Boulder, Colorado; Canton, Ohio; Chicago, Illinois; Cincinnati, Ohio; Cleveland, Ohio; Colorado Springs, Colorado; Columbus, Ohio; Denver, Colorado; Detroit, Michigan; Honolulu, Hawaii; Houston, Texas; Minneapolis-St.Paul, Minnesota; Nashville, Tennessee; New Haven, Connecticut; Pittsburgh, Pennsylvania; Provo-Orem, Utah; Salt Lake City, Utah; Seattle, Washington; Steubenville, Ohio; and Youngstown, Ohio.

For most cities, the metropolitan county encompassed the city and much of its suburbs, but we used multiple counties for Minneapolis-St. Paul (Ramsey and Hennepin), Birmingham (Blount, Jefferson, St. Clair, Shelby, and Walker), Steubenville (Jefferson, OH; Brooke and Hancock, WV), and Youngstown (Columbiana and Mahoning).

For each subject and follow-up period we created yearly averages (January–December) of pollution for that year and lags up to the 3 previous years.

### Analytical strategy

We defined the cohort as follows: We assumed that a subject admitted for MI enters the cohort if he survived at least 3 months and/or is alive the first January of the year following the admission. For each subject, the follow-up periods were 1 year periods (January–December) until the year in which they die (or suffer a subsequent MI or CHF admission for those analyses) or until December 1999 (censoring).

City-specific cohorts were created for the three survival analyses, one where failure was defined as death, one where failure was defined as a new MI, and one where failure was defined as a first admission for CHF.

We analyzed the data with an extended Cox’s proportional hazard regression model, which allows for time-varying covariates in survival analysis (Kleinbaum and Klein 2005). The model for the hazard *h* at time *t* is


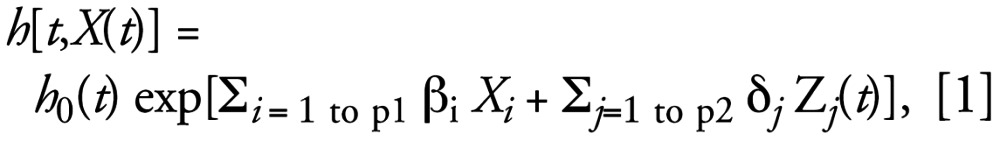


where *t* is time since a subject entered the cohort (January) after the admission for MI and is represented by 1 year; *X*_1_, … *X*_p1_ are time-invariant variables such as sex; and *Z*_1_(*t*),…, *Z*_p2_(*t*) are time-varying variables such as air pollution.

We adjusted for individual risk factors including age, sex, race, type of MI, number of days of coronary care and intensive care, previous diagnoses for atrial fibrillation, and secondary or previous diagnoses for COPD, diabetes, and hypertension, and for season of initial event as cold (December–February), hot (June–August), and transitional. To allow for possible nonproportionality of the survival rates, time period (three categories: 1985–1989–1990–1994, and 1995–1999), age (5-year categories), sex, race (white, black, others), and type of MI (10 categories) were treated as stratification variables.

Ties were treated using the approach of Kalbfleisch and Prentice (1980).

As a sensitivity analysis, we considered an alternative definition of our follow-up period. We defined yearly follow-up (and exposure averages) using a 12-month period starting the month of their index admission. We continued to construct 12-month average PM exposure for each subject for each subsequent year of follow-up (using month of initial event as the anniversary) until censoring or failure. In the last follow-up period for each subject, the person time at risk was < 12 months, and this was incorporated in the model. However, PM exposure was kept as a 12-month average to maintain comparability with other periods. When the last follow-up period was ≤ 3 months, subjects were censored at their last complete 12-month period of follow-up because the exposure interval was judged too short to be comparable to the 12-month exposure used for the follow-up periods.

We also restricted the mortality and CHF cohorts to subjects with a second MI, with follow-up beginning after the occurrence of their second MI, or including only those subjects who were admitted for their primary MI between 1985 and 1996, allowing at least 3 years of follow-up to all subjects in the analysis.

For each subject in each follow-up period, we considered the following possible exposure indexes: *a*) the average PM_10_ in their city in that follow-up period; and *b*) a model containing simultaneously the exposure during the follow-up period and each of the three previous years (distributed lag), to see if we could determine how the PM dropped off over time.

We first performed city-specific analyses; in the second stage of the analysis, the results were combined using the meta-regression technique of Berkey et al. (1998). To be conservative, we report the results incorporating a random effect, whether or not there was a significant heterogeneity.

Effect modification by individual risk factors was examined by fitting separate proportionate hazard models for each group (e.g., sex) in each city, controlling for covariates and combining across cities as in the main analysis. In addition, we examined effect modification by city characteristics by entering them as predictor variables in the meta-regression. These included measures of socioeconomic condition (percent in poverty), exposure-related measures (mean and interquartile range of PM_10_ in the city), general social factors (population density), and the emphysema death rate in persons ≥ 65 years of age as a surrogate for the smoking history of the population. The results are expressed as hazard ratio (HR) for 10 μg/m^3^ PM_10_.

## Results

There were 196,131 eligible MIs in the 21 cities during the study period. Table 1 shows characteristics of the study population for all the cities. Of the population, 45.5% died by the end of follow-up, 17% had a CHF admission after the index MI, and 11.5% had a subsequent MI. In the cohort, 63% of the subjects were ≥ 75 years of age. The most common types of MI were sub-endocardial infarction (34%), MI of other inferior wall (23%), and MI of other anterior wall (19%).

The average duration of the follow-up was 5.1 years for mortality, 3.7 years for CHF, and 3.6 years for subsequent MI. The range of survival times in all the cohorts varied from 1 to 14 years.

Table 2 presents city-specific characteristics, including the total population, PM concentrations, counts of hospital admissions for MI, and the numbers of deaths, subsequent acute MI, and first hospitalizations for CHF. The average PM_10_ across all cities was 28.8 μg/m^3^.

Table 3 presents the city-specific incidence rates for the three outcomes. In total, the incidence rates were 0.091 for death, 0.054 for CHF admission, and 0.027 for subsequent MI. The incidence rates among all cities were examined by year (Table 4); these were higher in the first 5 years but changed little during the following years, justifying the use of three categories to describe the time period.

We found significant associations in the three survival analyses adjusting for confounders (Table 5), with a hazard ratio for the sum of the distributed lag for mortality of 1.3 [95% confidence interval (CI), 1.2–1.5] per 10μg/m^3^ PM_10_, a hazard ratio of 1.4 (95% CI, 1.2–1.7) for CHF, and a hazard ratio of 1.4 (95% CI, 1.1–1.8) per 10 μg/m^3^ PM_10_ for a new hospitalization for MI. The distributed lag model shows greater effects at lags 1 and 2 exposure, with an overall effect considerably larger than for a single year.

Table 6 presents the results of the sensitivity analyses. When we restricted the mortality and CHF analysis to subjects with a second MI with follow-up beginning after the occurrence of their second MI, the effect of PM_10_ for the distributed lag model showed an HR of 1.3 (95% CI, 1.15–1.55) in the mortality cohort and an HR of 1.4 (95% CI, 1.22–1.65) in the CHF cohort. Including only those subjects who were admitted for their primary MI between 1985 and 1996, that is, with at least 3 years of follow-up, we found higher estimates than the main results reported in Table 5.

In Table 6 we also present the result of the sensitivity analysis in which we modified the definition of the cohort. For the distributed lag, we found an HR for mortality of 1.3 (95% CI, 1.15–1.4) per 10 μg/m^3^ PM_10_; this HR is similar to the main result in Table 5.

**Figure 1:**
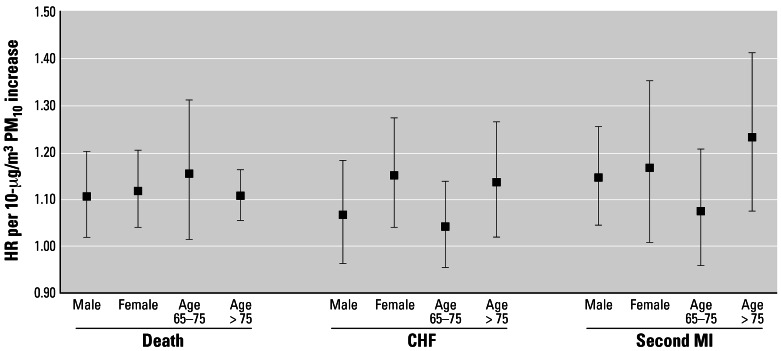
Effect modification by sex and age group in the three survival analyses, in which failure is defined as death, a subsequent MI, or CHF. The effects were computed by stratified analysis for each sex or age group (65–75 years or > 75 years) and PM_10_ averaged over 3 years. Effects are computed for 10 μg/m^3^ PM_10_.

Figure 1 shows the results of the analysis of effect modification by sex and age groups (65–75 years of age and ≥ 76 years). We did not find effect modification by sex, but we did find a higher effect in the older age group. Following the method of Payton et al. (2003) to determine whether the difference between the age groups was significant, we found a *p*-value for mortality of 0.064, whereas the *p*-value for subsequent MI and CHF was 0.082.

We used meta-regression (Berkey et al. 1998) to examine predictors of heterogeneity across city (Table 7), and we found that most of the predictors were not significant as modifiers of the PM_10_ effect.

## Discussion

We found a significant effect of long-term exposure to airborne particles on the risk of death, progression to heart failure, and a subsequent MI in a large multicity study of subjects discharged alive following an acute MI. This association was not due to differences between cities in exposure, but resulted from the association of year-to-year changes in mortality risk with year-to-year changes in exposure. We found that association persisted for several years of lag, but was falling off by lag 3.

Although several previous studies have reported an association of PM with mortality in survival analysis, this is the first long-term study that investigated persons discharged alive following an acute MI and showed that persons who survive an MI are at risk of death in response to subsequent particle exposure. The present study is the first large cohort study focused on the elderly.

One key difference between the present study and previous cohorts comes from the source of variation in exposure. In the other cohort studies, the source of exposure variation is across geographic area. For example, the ACS study (Pope et al. 2004a) contrasted covariate-adjusted survival in each city with long-term average pollution in that city. Using such an approach, unmeasured factors that vary across city are potential confounders. For example, substantial geographic variability in the use of cardiovascular medication has been reported in a number of studies, and this was not controlled in previous cohort studies. In the present study the basic analysis was conducted within each city, and exposure variation comes from temporal changes in pollution concentration. This eliminates those potential confounders as a concern. By focusing on 12-month average exposures, it also eliminates the potential confounding by short-term weather factors that are an issue in time-series studies. Obviously, factors that fluctuate from year to year within each city are potential confounders in this study design. The advantage of this approach is that it allows us to use an analytical methodology with different vulnerabilities to confounding than in previous studies; to determine whether an association between PM and mortality risk persists; and to examine an intermediate time period of exposure—in contrast to the use of daily exposure in time-series studies—and exposure over many years in other cohorts.

The use of longitudinal rather than cross-sectional exposure gradients in this study may also explain some of the differences in effect size estimates, because the variation in central station monitoring and personal exposure over time may be more correlated than similar variations over space. Moreover, the ACS study (Pope et al. 2004a) used monitors within the multicounty metropolitan areas to assign exposure, whereas our subjects are matched to monitors in the same city or county. A recent reanalysis of ACS data restricting to persons living in the same county of the monitor reported a larger risk (Pope et al. 2004a; Willis et al. 2003).

The sensitivity analysis in the present study showed that subjects with a second MI have a higher risk of a PM-associated subsequent death. Hence, this appears to represent a particularly susceptible group.

In a recent case–crossover study examining this association, Bateson and Schwartz (2004) reported much smaller relative risks (1.02; 95% CI, 0.99–1.04). In another case–crossover study (Zanobetti and Schwartz 2005) on 21 U.S. cities, we analyzed the short-term effect of PM_10_ on the MI hospitalization in these cities. In that analysis, we also found a smaller relative risk (1.007; 95% CI, 1.003–1.01). Although part of the association reported here may be acute, the evidence indicates cumulative exposure over a year or more elevates risk above and beyond the effects of acute exposure.

We observed a high degree of heterogeneity among the cities. In multicities time-series studies of short-term effect of air pollution on health, heterogeneity has been found among cities (Katsouyanni et al. 2001; Le Tertre et al. 2002; Samet et al. 2000). Heterogeneity has been attributed to, for example, differences in particle characteristics, ventilation rates of buildings, average PM_10_ concentrations, and social conditions. Previously published cohort studies could not address the issue of heterogeneity because the studies were essentially cross-sectional; the source of exposure variation was across geographic area. This is the first long-term study to show significant heterogeneity among cities in response to long-term exposure, and the cause of this heterogeneity needs to be determined.

This analysis advances the field *a*) by reporting an association between particle exposure and survival in a cohort study of MI survivors that eliminates geographic variation in risk factors as a confounder, but where exposure variation comes from temporal changes in pollution concentration; and *b*) by focusing on associations on an intermediate time scale. If PM increases progression of atherosclerosis (Künzli et al. 2005; Suwa et al. 2002) or impairs endothelial function (Brook et al. 2004) or autonomic function (Liao et al. 1999; O’Neill et al. 2005), these changes may have greater impact in populations with greater underlying impairment, such as the elderly.

A primary candidate for explaining these risks must be the acute MI itself (Berger et al. 1992; Guidry et al. 1999; Kannel et al. 1979). Subjects surviving MIs have enhanced risk of dying and decreased heart rate variability (Malik et al. 1989), and are likely to have greater susceptibility to subsequent insults.

This possibility of multiple pathways to mortality makes it plausible that the all-cause mortality risk might exceed the risk of specific cardiac events, as we observe for subsequent MI. For example, PM has been associated with arrhythmias (Peters et al. 2000), pneumonia (Zanobetti et al. 2000), and COPD (Schwartz 1993), creating potential additional pathways by which exposure could increase mortality risk independent of MI risk.

One limitation of the present study is that Medicare does not provide the underlying cause of death. If the cause of death were available, we could understand better the possible pathways.

Future studies examining cohorts with more detailed clinical data on the MI survivors should be fruitful. The increased risk of heart failure following the MI also suggests that further evaluation of this outcome is warranted.

The present study presents additional limitations, the main one being the absence of information on subject characteristics such as smoking, body mass index, or medicine use, and information on whether the patients migrated out of the study areas after their last Medicare contact. However, we were able to adjust for other important characteristics such as age, race, sex, the type of MI, and previous and secondary diagnoses. Missing those other characteristics would only confound the association with air pollution if they were correlated with pollution. However, we conducted a city-specific analysis to remove location-specific differences in the analyses. Hence, differences across cities in smoking rates, for example, cannot confound the association, because only the temporal variability in pollution within city contributes to the association. Smoking could only confound this association if year-to-year variations in smoking rates within city covaried with year-to-year variations in PM_10_ concentrations. Moreover, we examined the emphysema death rate in persons > 65 years of age as an effect modifier because it is associated with smoking history in the population, and found that it did not modify the PM-associated risk. Thus, smoking is unlikely to be a confounder in our study.

Our findings that both subsequent MIs and CHF admissions, as well as mortality risk, are elevated suggests that multiple pathways are involved in the particle effects.

Other human and animal studies (Godleski et al. 2000) have shown associations between particulate pollution and changes in heart rate variability (Gold et al. 2000; Liao et al. 1999; Pope et al. 1999); increases in plasma viscosity (Peters et al. 1997), C-reactive protein (Brook et al. 2003; Peters et al. 2001b; Pope et al. 2004b), plasma fibrinogen (Ghio et al. 2000), white blood cell counts (Salvi et al. 1999; Schwartz 2001), blood pressure (Ibald-Mulli et al. 2001; Linn et al. 1999; Zanobetti et al. 2004), and oxidative stress (Bouthillier et al. 1998; Brook et al. 2003; Sorensen et al. 2003); decreases in plaque stability (Suwa et al. 2002); or occurrence of thrombotic complications after exposure to pollutants (Nemmar et al. 2003). In a recent study O’Neill et al. (2005) reported that PM was associated with flow-mediated dilation of the brachial arteries.

Many of these associations are with acute exposure, not long-term exposures such as those used in the present study. Nevertheless, the associations suggest that the hypotheses to explain the potential mechanisms for the particle effects might involve systemic inflammation, changes in autonomic function, or oxidative stress capable of influencing both cardiovascular and pulmonary physiology.

Our findings suggest that persons surviving an MI are at risk from exposure to particulate pollution. This is a large group, and hence this finding has substantial public health implications. Our results also suggest that it would be beneficial to examine this population in mechanistic studies.

## Figures and Tables

**Table 1:** Characteristics of the study population among residents of 21 U.S. cities.

Characteristic	No. of events	Percent	Mean (5th–95th percentiles)
MI	196,131		
Failure			
Deaths	89,249	45.5	
CHF	33,764	17.2	
Subsequent MI	22,552	11.5	
Baseline characteristics			
Age			76.1 (66.5–89.1)
Sex			
Male	98,822	50.4	
Female	97,309	49.6	
Race			
White	165,549	84.4	
Black	19,759	10.1	
Other	10,823	5.5	
No. of days in coronary care			1.3 (0–6.5)
No. of days in intensive care			1.5 (0–6.5)
Type of MI			
Anterolateral wall	10,088	5.1	
Other anterior wall	37,993	19.4	
Inferolateral wall	6,434	3.3	
Inferoposterior wall	4,291	2.2	
Other inferior wall	44,923	22.9	
Other lateral wall	5,326	2.7	
True posterior wall infarction	2,009	1.0	
Subendocardial infarction	66,628	34.0	
Other specified sites	3,951	2.0	
Unspecified sites	14,488	7.4	
Secondary or previous diagnoses			
COPD	28,509	14.5	
Diabetes	44,686	22.8	
Hypertension	72,154	36.8	
Previous admissions			
Atrial fibrillation	11,374	5.8	

**Table 2:** City-specific counts of hospital admissions for MI, second MI, first CHF, and deaths and for distribution of PM_10_.

						PM_10_^a^ (%)		
City	MI dmissions	Deaths	CHF events	2nd MI events	No. ≥ 65 years of age^b^	10	50	90
Birmingham, AL	8,927	4,281	1,814	1,028	120	22.8	27.0	38.2
Boulder, CO	1,117	434	176	97	17	18.0	20.6	28.9
Canton, OH	4,788	2,061	797	594	53	22.1	25.2	28.4
Chicago, IL	42,091	20,333	7,673	5,130	632	29.5	33.4	38.5
Cincinnati, OH	7,961	3,778	1,381	906	115	25.5	30.7	38.2
Cleveland, OH	16,648	7,767	3,140	1,899	221	35.2	37.6	42.5
Colorado Springs, CO	2,054	672	282	173	32	18.4	21.0	24.9
Columbus, OH	7,859	3,574	1,370	1,044	92	25.6	28.5	31.6
Denver, CO	3,919	1,551	546	357	64	26.6	28.9	36.5
Detroit, MI	18,437	8,490	3,439	2,098	264	28.0	31.3	37.9
Honolulu, HI	4,528	1,952	633	484	91	14.8	16.3	18.7
Houston, TX	10,885	4,764	1,812	975	196	26.0	29.7	32.3
Minneapolis, MN	10,123	3,962	1,360	1,019	176	22.3	24.7	31.8
Nashville, TN	4,081	1,877	697	449	59	27.1	30.2	38.5
New Haven, CT	9,232	4,245	1,447	1,119	118	22.2	24.0	29.3
Pittsburg, PA	20,663	10,007	3,653	2,800	233	25.2	29.5	34.3
Provo/Orem, UT	1,504	535	258	136	18	26.3	32.4	38.5
Salt Lake City, UT	3,535	1,233	425	256	61	28.2	34.1	43.3
Seattle, WA	9,674	4,011	1,325	921	167	16.0	22.6	31.7
Steubenville, OH	2,502	1,130	499	302	24	26.4	33.9	37.7
Youngstown, OH	5,603	2,592	1,037	765	61	27.2	29.2	33.3

a Distribution of the individually assigned 1-year PM10 mean in each city.

b Population ≥ 65 years of age (× 1,000).

**Table 3:** Accrued person-time and incidence rate for the three survival analyses.

	Person-years			Incidence rate		
City	Deaths	CHF	2nd MI	Deaths	CHF	2nd MI
Birmingham, AL	44,672	28,995	39,495	0.120	0.063	0.026
Boulder, CO	6,292	4,014	5,305	0.084	0.044	0.018
Canton, OH	23,826	15,333	20,336	0.108	0.052	0.029
Chicago, IL	207,151	125,216	180,216	0.123	0.061	0.028
Cincinnati, OH	39,967	25,530	35,255	0.118	0.054	0.026
Cleveland, OH	83,790	52,392	72,310	0.116	0.060	0.026
Colorado Springs, CO	10,475	7,813	9,430	0.080	0.036	0.018
Columbus, OH	37,725	24,977	33,535	0.120	0.055	0.031
Denver, CO	22,424	15,187	20,178	0.084	0.036	0.018
Detroit, MI	87,612	55,024	74,111	0.123	0.063	0.028
Honolulu, HI	21,831	14,842	19,632	0.113	0.043	0.025
Houston, TX	52,910	36,734	46,250	0.113	0.049	0.021
Minneapolis, MN	51,318	34,124	46,421	0.096	0.040	0.022
Nashville, TN	20,710	14,039	18,168	0.113	0.050	0.025
New Haven, CT	46,935	28,516	40,926	0.113	0.051	0.027
Pittsburg, PA	101,145	63,399	82,726	0.124	0.058	0.034
Provo/Orem, UT	8,138	5,401	6,798	0.081	0.048	0.020
Salt Lake City, UT	19,021	13,236	17,136	0.080	0.032	0.015
Seattle, WA	52,168	34,519	46,465	0.094	0.038	0.020
Steubenville, OH	11,674	6,986	9,311	0.123	0.071	0.032
Youngstown, OH	27,146	16,902	23,182	0.120	0.061	0.033
Total	976,930	623,179	847,186	0.091	0.054	0.027

**Table 4:** Accrued person-years, number of deaths, and incidence rate (IR) across all cities by year for the mortality cohort.

Year	Person-years	No. of deaths	IR
1986	10,015	1,423	0.142
1987	22,094	2,808	0.127
1988	32,640	3,675	0.113
1989	41,852	4,173	0.100
1990	50,173	4,786	0.095
1991	59,419	5,581	0.094
1992	67,713	6,223	0.092
1993	76,769	6,711	0.087
1994	85,574	7,709	0.090
1995	93,209	8,427	0.090
1996	100,260	8,803	0.088
1997	107,150	9,152	0.085
1998	113,019	9,617	0.085
1999	117,043	10,161	0.087

**Table 5:** HR and 95% CI for 10-μg/m^3^ increase in PM_10_ for the year of failure and for the distributed lag from the year of failure up to 3 previous years.

Failure	HR	95% CI	*p*-Values
Death			
PM_10_ annual	1.11	1.05–1.19	0.001
Distributed lag model			
Lag 0	1.04	0.96–1.14	0.336
Lag 1	1.07	0.99–1.14	0.070
Lag 2	1.14	1.10–1.18	0.000
Lag 3	1.06	0.99–1.12	0.077
Sum lags 0–3	1.34	1.17–1.52	0.000
CHF			
PM_10_ annual	1.11	1.03–1.21	0.009
Distributed lag model			
Lag 0	1.09	1.01–1.18	0.030
Lag 1	1.09	1.01–1.19	0.038
Lag 2	1.13	1.02–1.25	0.014
Lag 3	1.04	0.97–1.12	0.260
Sum lags 0–3	1.41	1.19–1.66	0.000
Second MI			
PM_10_ annual	1.17	1.05–1.31	0.003
Distributed lag model			
Lag 0	1.09	0.92–1.30	0.325
Lag 1	1.12	0.97–1.30	0.108
Lag 2	1.15	1.08–1.23	0.000
Lag 3	1.01	0.94–1.09	0.783
Sum lags 0–3	1.43	1.12–1.82	0.005

Models controlled for season, days of coronary care and intensive care, previous diagnosis for atrial fibrillation, and secondary or previous diagnoses for COPD, diabetes, and hypertension; we adjusted for time period, age, sex, race, and type of MI as stratification variables.

**Table 6:** HR and 95% CI for 10 μg/m^3^ increase in PM_10_ (sum of previous 3 years distributed lag) for the sensitivity analyses.

	HR	95% CI	*p*-Values
Death			
Subjects with subsequent MI^a^	1.33	1.15–1.55	0.000
Subjects admitted between 1985 and 1996^b^	1.45	1.26–1.68	0.000
Second definition of cohort^c^	1.29	1.15–1.44	0.000
CHF			
Subjects with subsequent MI^a^	1.42	1.22–1.65	0.000
Subjects admitted between 1985 and 1996^b^	1.51	1.26–1.81	0.000
Subsequent MI			
Subjects admitted between 1985 and 1996^b^	1.62	1.23–2.13	0.001

a Follow-up started after subsequent MI.

b Includes only primary admission for MI during 1985 and 1996.

c Yearly follow-up and 12-month average PM_10_ exposure for each subject for each subsequent year of follow-up starting from the month of the index admission until censoring or failure.

**Table 7:** Modification of the PM_10_ association in the three survival analyses by city characteristics across 21 U.S. cities expressed as HR and 95% CI for 10-μg/m^3^ increase in PM_10_ (distributed lag) estimated at the 25th percentile and the 75th percentile of the effect modifier.

		HR at the 25% percentile			HR at the 75% percentile		
City characteristic	*p*-Value for modifier	1st quartile	HR	95% CI	3rd quartile	HR	95% CI
Population ≥ 65 years of age in poverty status (%)							
Death	0.60	8.0	1.34	1.06–1.70	11.4	1.27	1.05–1.53
CHF	0.83		1.36	1.05–1.75		1.32	1.08–1.62
MI	0.61		1.29	0.91–1.82		1.19	0.91–1.56
Annual mortality rate for emphysema ≥ 65 years of age							
Death	0.57	32.9	1.33	1.07–1.67	47.6	1.27	1.04–1.54
CHF	0.37		1.42	1.12–1.79		1.30	1.06–1.60
MI	0.97		1.26	0.91–1.73		1.25	0.94–1.66
Mean PM_10_							
Death	0.60	25.5	1.25	1.01–1.55	32.1	1.33	1.07–1.65
CHF	0.74		1.36	1.08–1.72		1.30	1.03–1.64
MI	0.04		1.02	0.76–1.37		1.43	1.08–1.91
IQR PM_10_							
Death	0.70	3.3	1.31	1.07–1.62	5.5	1.26	1.02–1.56
CHF	0.38		1.40	1.12–1.74		1.26	1.01–1.58
MI	0.28		1.12	0.83–1.50		1.33	0.98–1.79
Population density							
Death	0.67	594.8	1.33	1.06–1.67	2076.9	1.27	1.05–1.54
CHF	0.75		1.30	1.02–1.66		1.35	1.10–1.65
MI	0.74		1.17	0.84–1.64		1.23	0.93–1.62

IQR, interquartile range.
